# High seroprevalence of COVID-19 infection in a large slum in South India; what does it tell us about managing a pandemic and beyond?

**DOI:** 10.1017/S0950268821000273

**Published:** 2021-02-04

**Authors:** Carolin Elizabeth George, Leeberk Raja Inbaraj, Sindhulina Chandrasingh, L. P. de Witte

**Affiliations:** 1 Division of Community Health and Family Medicine, Bangalore Baptist Hospital, Bangalore 560024, Karnataka, India; 2 Department of Microbiology, Bangalore Baptist Hospital, Bangalore 560024, Karnataka, India; 3 School of Health and Related Research (ScHARR), University of Sheffield, Sheffield, UK

**Keywords:** COVID-19, Covid-19 in slum, India, SARS CoV2, seroprevalence

## Abstract

People living in urban slums or informal settlements are among the most vulnerable communities, highly susceptible to coronavirus disease 2019 (COVID-19) infection and vulnerable to the consequences of the measures taken to control the spread of the virus. Fear and stigma related to infection, mistrust between officials and the population, the often-asymptomatic nature of the disease is likely to lead to under-reporting. We conducted a cross-sectional study to determine the seroprevalence of COVID-19 infection in a large slum in South India 3 months after the index case and recruited 499 adults (age >18 years). The majority (74.3%) were females and about one-third of the population reported comorbidities. The overall seroprevalence of IgG antibody for COVID-19 was 57.9% (95% CI 53.4–62.3). Age, education, occupation and the presence of reported comorbidities were not associated with seroprevalence (*P*-value >0.05). Case-to-undetected-infections ratio was 1:195 and infection fatality rate was calculated as 2.94 per 10 000 infections. We estimated seroprevalence of COVID-19 was very high in our study population. The focus in this slum should shift from infection prevention to managing the indirect consequences of the pandemic. We recommend seroprevalence studies in such settings before vaccination to identify the vulnerability of COVID-19 infection to optimise the use of insufficient resources. It is a wake-up call to societies and nations, to dedicate paramount attention to slums into recovery and beyond – to build, restore and maintain health equity for the ‘Health and wellbeing of all’.

## Introduction

Coronavirus disease 2019 (COVID-19) is a pandemic of historic significance characterised by its ubiquitous presence, accelerated expansion and catastrophic economic consequences. Globally, over a few months of the pandemic outbreak, India became one of the epicentres of this contagion, contributing to one-sixth of the world's reported cases [1]. ‘Slums’ or ‘informal settlements’ which are home to at least 5.4% (65.5 million) of India's population, paint the most vulnerable landscape for COVID-19 infections, both in terms of susceptibility and consequences [2].

**Fig. 1.**
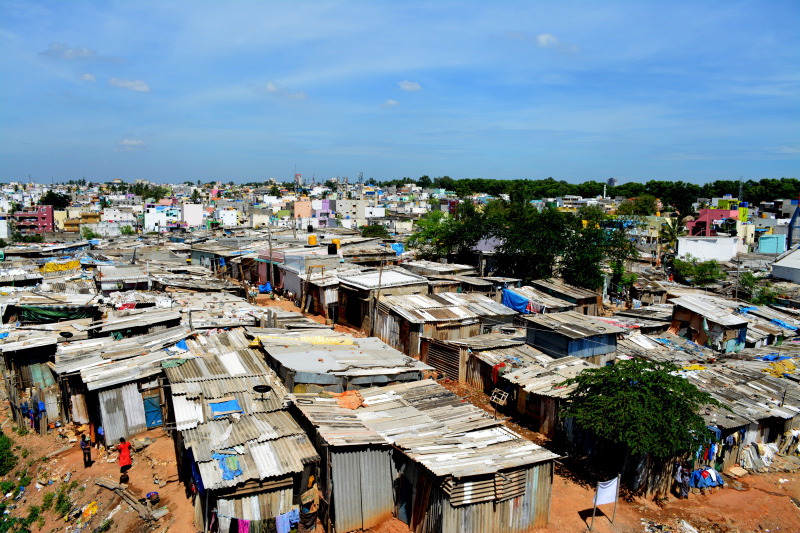
Aerial View of Devarajeevanahalli (DJ Halli) slum, Bangalore [1].

Devarajeevanahalli (DJ Halli), also known as the ‘Dharavi of Bangalore’ is one of the largest governments notified slums in Bangalore, extending over 1.15 km^2^ with an estimated population of 100 000 people [3–5] (Fig. 1). The Bangalore Baptist Hospital (BBH) has been rendering primary care services in this slum for the past decade. BBH supported the DJ Halli population and complemented government efforts by continuing primary health services throughout the pandemic when all other private hospitals in the area closed owing to high risk of infection in slums [6, 7]. Because DJ Halli is a typical slum with a likelihood of rapid contagion, the official data of less than 295 cases and 17 deaths, 5 months past the first case, sounded unrealistic [8, 9]. Poor cooperation with the state government's testing efforts was a reality as the people in DJ Halli did not trust the government's welfare motives after parliament passed the Citizenship Amendment Act, 2019 [10, 11]. Further, screening and testing efforts were hindered by communal violence which claimed three lives in this area [12].

At this time, officials, public health experts and healthcare providers were not clear about the infection status of the slum. One side argued that effective campaigns curtailed infection in the slums and the other group postulated high rate of asymptomatic infection. Both arguments were plausible and needed different resource allocation: for low infection rate and high susceptibility, our response should be COVID-19 specific and for high infection and herd immunity, our response should gear towards resource re-allocation to other diseases and damages caused by COVID-19 crisis.

Though challenging, investigating the seroprevalence of COVID-19 in DJ Halli slum was critical for us to plan the health and social interventions in this slum. Since there is a lack of information regarding the seroprevalence in slums, the study will add to the body of evidence – to help city health officials locally and public health scientists globally.

The objective of the current study was to estimate the seroprevalence of COVID-19 infection in a dense slum of South India. We hypothesised a high prevalence of infection, considering the density of the population and the impossibility of preventive measures in this setting.

## Materials and methods

### Study design and sample size

We designed a cross-sectional seroepidemiological survey in DJ Halli slum based on the recommendation of WHO as the most appropriate study design after the peak transmission is established [13]. A study from slums in Mumbai, India, has reported a seroprevalence of 57% [14]. We used the formulae 4 *Pq*/*d*^2^ and calculated a minimum sample size of 470 with an absolute precision of 5% and a design effect of 1.2.

### Data collection

DJ Halli consists of two wards (administrative blocks), each of which was further divided into four clusters for sampling purposes. A kiosk was set up in one of the trusted community spaces in each cluster. Our community health workers invited people (adults ⩾18 years) from houses to give blood samples. If a household refused to participate, then the next house was approached. In each cluster, mobilisation continued till the desired sample size was achieved. We explained the purpose of the study, took written consent and interviewed people with a questionnaire. The questionnaire contained questions about demographic information (age, gender, education, comorbidities such as diabetes, hypertension, lung disease and cancer), history of exposure to COVID-19 infection (history of being diagnosed as COVID-19 case, interaction and household contacts with persons with confirmed COVID-19), any history of COVID-19-related symptoms a month before the survey and their self-reported compliance to handwashing and use of face masks. Our Phlebotomists collected 5 ml of blood from each participant via venepuncture in a plain vacutainer and transported to BBH laboratory (The Indian Council of Medical Research (ICMR) and NABL (National Accreditation Board for Testing and Calibration Laboratories) accredited) within 5 h maintaining the cold chain.

### Sample processing and analysis

The serum was separated and used to test for antibodies using the Elecsys Anti SARS CoV2 assay, an electrochemiluminescent immunoassay using a recombinant protein representing the nucleocapsid (N) antigen for the determination of high-affinity antibodies including IgG against SARS CoV2 [15]. This assay employs a cutoff index (COI) that is automatically calculated from two calibration standards – a COI of 1.0 or more is considered reactive/positive and a COI less than or equal to 1.0 is reported as nonreactive/negative. Serum samples with indeterminate results were repeat tested and on with indeterminate results on repeat testing were considered as negative. The assay sensitivity and specificity were reported to be 97.2% (95.4–98.4) and 99.8% (99.3–100), respectively, in samples taken 30 days or more post-symptom onset [16].

### Statistical analysis

The frequency of characteristics of the survey participants was described. Seroprevalence of COVID-19 IgG antibody was reported in per cent with 95% confidence interval (CI). Case-to-undetected-infections ratio (CIR), was calculated as a ratio of the number of reported quantitative real-time polymerase chain reaction (RT-qPCR)-confirmed COVID-19 cases, 2 weeks before the initiation of serosurvey (IgG antibodies against SARS-CoV-2 infection start appearing by the end of the first week after symptom onset and most cases are IgG positive by the end of the second week) to the number of people who have IgG antibodies [17]. Assuming a 3-week lag time from infection to death, we considered the reported number of deaths after 3 weeks of the survey to estimate the plausible range of the infection fatality ratio (IFR) [16]. It was calculated as the number of deaths reported upon the total number of people with IgG antibodies per 10 000 infections. The association of seroprevalence with comorbid conditions and socio-demographic characteristics was tested using chi-square tests.

### Ethical consideration

The Ethics Committee on Bangalore Baptist Hospital approved the survey protocol on 30 June 2020. Written informed consent was obtained from the participants and the test results were communicated to them.

## Results

Our 499 participants were equally distributed in both wards (0.53% and 0.47%). The mean age was 39.7 + / 14.5 years and the majority (74.3%) were females. Most people (96.9%) had less than 12 years of education and 56.5% did not have any job. About one-third of the population reported comorbidities (see Table 1). Hypertension (19.2%) and diabetes (15.4%) were reported as the most common comorbidities. The majority of people reported frequent use of hand sanitisers (97.8%) and face covers (88.4%).

**Table 1.** Socio-demographic profile of the study population

Demographics	Categories	*N*	%
Age (in years)	≤20	36	7.2
	21–40	254	50.9
	41–60	167	33.5
	>60	42	8.4
Gender	Male	128	25.7
	Female	371	74.3
Education	Illiterate	126	25.3
	Primary to high school (⩽12 years of formal education)	357	71.6
	Graduate	16	3.1
Occupation	Unemployed	282	56.5
	Domestic helper	38	7.6
	Daily wage labourer	55	11
	Professional	53	10.6
	Others	71	14.2
Comorbidities	Any comorbidities	159	31.9
	Diabetes	77	15.4
	Hypertension	96	19.2
	Heart diseases	5	1.0
	Others	20	4;0
Risk factors	Tested positive for COVID 19	15	3.0
	Family member tested positive for COVID 19	12	2.4
	Had an interaction with COVID 19 patient	9	1.8

The overall seroprevalence of IgG antibody for COVID-19 was 57.9% (95% CI 53.4–62.3) (Table 2). When we adjusted for the sensitivity and specificity of the diagnostic kit the adjusted seroprevalence was 54.6% (95% CI 50.23–58.97). Seroprevalence among participants with diabetes and hypertension was 62.3% and 66.6%, respectively, but the association with seropositivity was not significant. Among the seropositive individuals, 41.6% had a history of an infected family member and 33.3% gave a history exposure to a COVID-19 infected person in the past. The majority (95.2%) of the seropositive individuals, did not report any symptom related to COVID-19 infection at the time of the study nor in the past. This study estimated 195 undetected infected individuals for every RT-PCR confirmed case, i.e. CIR of 1:195. The IFR was calculated as 2.94 per 10 000 infections as on 20 October 2020 in this slum.

**Table 2.** COVID-19 seroprevalence in the study population

	Category	Male	Prevalence (95% CI)	Female	Prevalence (95% CI)	Total	Overall prevalence (95% CI)
Age (years)	≤20	13	46.2 (19.2–74.9)	23	56.5 (34.5–76.8)	36	52.8 (35.5–69.6)
	21–40	55	43.6 (30.3–57.0)	199	61.8 (54.7–68.6)	254	57.9 (51.5–64.0)
	41–60	42	54.8 (38.7–70.2)	125	61.6 (52.5–70.2)	167	59.9 (52.0–67.4)
	>60	18	44.4 (21.5–69.2)	24	62.5 (40.6–81.2)	42	54.8 (38.7–70.2)

Age, education, occupation, presence of comorbidities and presence of self-reported symptoms were not associated with seroprevalence (*P*-value >0.05) whereas female gender was significantly associated with seroprevalence.

## Discussion

Our findings suggest very high COVID-19 seroprevalence in DJ Halli slum. This is consistent with the study from Mumbai slums, where a prevalence of 57% was noted 3 months past index case. If we extrapolate our findings to the whole of DJ Halli slum, 57 900 people in this slum would have contracted the infection in contrast to 295 cases which were reported. This is no surprise, as the hazardous physical environment, overcrowding, poor sanitation and the impracticability of social distancing, hand washing and face covers in slums, are conducive for the rapid spread of infection [18–22]. Another important learning is that the vast majority of cases were asymptomatic. Hence, they harboured and dispersed infection efficiently without being caught by the tests or inviting medical attention.

A recent survey done in all 30 districts of Karnataka state, conducted from 16 September 2020 among all adults aged 18 years and above, reported adjusted IgG seroprevalence of 16.4% [23]. Though low (pregnant women attending the antenatal clinic), moderate (persons moving in the community) and high risk (elderly and persons with comorbid conditions) population group were covered, the survey did not include population from slums, a possible explanation for low prevalence. This study also estimated that there were 40 undetected infected individuals for every RT-PCR confirmed case, i.e. CIR of 1:40, ranged from 10 to 111 across units [23]. In the national seroprevalence survey conducted by ICMR, the CIR was 81.6–130.1 in the first round (May) [24]. Our CIR is higher than other surveys and can be attributed to the low testing rates at the beginning of the epidemic.

Calculation of IFR is dependent on an accurate reporting of deaths and the number of estimated infections. Reported IFR may be an underestimate, as the real death due to COVID 19 is likely to be more than reported deaths. The overall IFR based on the serosurvey findings was much higher than that reported from Santa Clara County, USA (0.12–0.2%), Iran (0.08–0.12%), Brazil and Spain (1%) and of India in May [24–26]. This can be explained by the differences in access to healthcare facilities, quality of care and variation in the prevalence of comorbidities

A previous study in the same slum noted a higher prevalence of hypertension (35.5%) and diabetes (16.6%) [8]. In that previous study, the health professionals measured blood pressure and blood sugar using a screening toolkit during a household survey. The study showed a reported-actual discrepancy in comorbidity prevalence, as more than half of the people with comorbidities were detected during the study. People in slums are known to have poor health-seeking behaviour; hence the actual proportion of people with comorbidities may be higher than what we reported in the current study.

The study participants reported improvement in hygiene measures like hand sanitation and frequent use of face covers. This may be due to improved behaviour following COVID-19 awareness campaigns led by the government and civic bodies and mandatory workplace enforcement. The possibility of social desirability bias (a discordance was noted as many were not wearing masks though they labelled themselves as frequent mask users during the survey) cannot be ruled out in this setting.

What does this ‘high seroprevalence’ mean to us? First and foremost, it suggests that the worst is over. It is a relief to realise that the virus spared most lives in this slum. With more than half of the population already being infected, the infection curve is likely to have started its journey down and COVID-19 will cease to be a public health problem in DJ Halli. This is confirmed by a low COVID-19 test positivity rate (1.5–2%) despite adequate testing (2000 tests per million/day) from this slum in the past one and half months (unpublished data from the government). Likewise, we also did not see any suspected cases from our DJ Halli clinic in the past 2 months. These findings provoke us to rethink the need for a vaccine in this slum. If half the population have antibodies, should we still call them vulnerable to COVID-19? Is any vaccine a match for natural infection in these settings? This study is not giving easy answers, but it does give rise to such questions.

Second, the findings suggest it is time to shift our strategy from chasing the virus' to ‘mending the damages’; damages caused by other diseases when COVID-19 annexed our undivided attention and the damage caused by the loss of livelihoods. During the pandemic, the entire health system was reorganised to support to pandemic response leading to scaling down and suspension of disease control programmes, immunisation and primary health care services. Due to fear, people postponed their hospital visits for their non-communicable diseases and other life-threatening conditions. Loss of jobs has aggravated hunger and nutritional deprivation. In a survey by the WHO among 105 countries around the globe, almost every country (90%) experienced a disruption to some extent, with greater disruptions being reported in low- and middle-income than in high-income countries in essential health services across the life course [27]. We need to be cognizant about the gravity of this situation in slums which struggled with poor healthcare networks even before the pandemic and its implications on public health in the coming months.

The pandemic was clearly a catch-22 situation in slums, where, if people continued their life, as usual, they faced the risk of getting infected. If they stayed at home as was being directed, they lost their livelihoods and sources of sustenance. Hence, we shall have to find ways to keep providing ‘regular’ healthcare to prevent the consequences of trying to control the virus being more damaging than the virus itself. This is especially relevant in settings like this slum, where it is practically impossible to prevent the spread of the virus.

The pandemic has also brought mercies. Slums have never experienced so much attention and care as during this pandemic. Every city highlighted their slums on their maps; we witnessed ‘missing millions’ becoming the ‘pinnacle of attention’. Government and civic bodies formed alliances, crafted strategies, pooled in resources to serve slums, thus attaining unbelievable progress on many domains in the past 3 months [28].

The possibility of another pandemic and the vulnerability of slums are stark realities in front of us. Had the mortality been higher, the contagion would have wiped millions and the slums would have been the reservoirs. Hence, we should take the lessons from this pandemic seriously. The first step would be recognising the slums and their inhabitants by improving basic living conditions, facilitating stable economic inclusion and promoting access to quality education and health services. Such investments in slums will reap huge dividends due to neighbourhood effects [29]. So, the COVID-19 pandemic is a wake-up call for governments to prioritise the humanity and dignity of residents of urban slums and to engage with these communities and experts to co-create solutions to promote the wellbeing of cities and its population.

The study may have some potential biases. We have selected different locations in the specific slum and then people were invited for the study. This sampling might have led to selection bias which could have impacted the true prevalence estimation. However, we were not able to assess the impact of this on the prevalence. Measurement bias could be another possibility due to validity parameters of the test used, however, we attempted to overcome this by reporting test performance adjusted seroprevalence rate with CI.

This is the first study reporting seroprevalence from a slum in South India and the second study from India. It corroborates the findings of the first study (peak attained within 3 months of index case) and strengthens the body of evidence related to one of the most vulnerable populations. It also gives guidance for the planners on allocating resources judiciously between COVID and non-COVID care in the slums of India which harbour more than 65 million people.

The study had few limitations. Firstly, the sampling strategy emphasised more on pragmatism than representativeness in the context of the communal violence which erupted in DJ Halli 2 weeks before the survey. Secondly, we have presented unadjusted seroprevalence rate as there was no data available on the age-sex distribution of this population. However, the estimates are very likely to be close to the real estimate as the prevalence was almost similar in all the strata. Thirdly, our sample had a smaller number of men compared to women as they were not available at home during the daytime. This might have resulted in an underestimation of seroprevalence as men have more social contacts than women in this context [24]. Fourthly, the study participants were interviewed to collect information about the history of the symptoms for the preceding month. However, as the presence of IgG antibodies reflects exposure to COVID 19 since the beginning of the pandemic, we were not able to estimate how many seropositive individuals ever had probable COVID-19 symptoms. Lastly, since we have not done RT-PCR or IgM antibodies separately, we would have missed people who were currently harbouring infection, resulting in a slight underestimation of the prevalence.

## Conclusion

The study in a dense slum in South India after 3 months of index case showed a high seroprevalence of COVID-19 infection in this setting. For every case reported, there were 195 undetected cases, which unearths the implication of the often-asymptomatic nature of the disease in reflecting the true count of people with COVID-19 infection. Vulnerability to infection is the primary condition to assign priority for COVID-19 vaccination, due to vaccine demand exceeding our existing capacity of vaccine production. Hence, we should keep in mind the possibility of most vulnerable communities achieving immunity with natural infection, thus negating the benefit of the vaccine in this population. The process of carrying out a seroprevalence study in dense settlements before vaccination may prove advantageous in identifying the disadvantaged communities which will benefit most from vaccination.

It is vital to realise that containing an infectious respiratory virus was practically impossible in slums, even with the best of efforts from all sectors. Slums received attention and coordinated efforts from government and civic societies during this pandemic, which is containing the damage caused by the pandemic. We should nurture these networks beyond the pandemic to strengthen health security of slums and their inhabitants.

Since the infection is controlled in slums like DJ Halli, what is the way forward – ‘hands-off’ or ‘all hands on the deck approach?’ The answer lies in the wisdom – that the debate should not only be about the virus but more importantly, about the people living in slums!

## Data Availability

The datasets used and/or analysed during the current study are available from the corresponding author on reasonable request.
